# Analysis of Treatment Strategies and Outcomes in Malignant Peritoneal Mesothelioma: Insights From a Multi-Center Study

**DOI:** 10.1245/s10434-024-15506-3

**Published:** 2024-05-28

**Authors:** Serkan Yaşar, Feride Yılmaz, Güngör Utkan, Efnan Algın, Doğan Bayram, Selim Tamam, Ömür Berna Çakmak Öksüzoğlu, Ayşegül İlhan, Efe Cem Erdat, Ali Ekrem Ünal, Şuayib Yalçın

**Affiliations:** 1 Department of Medical Oncology, Hacettepe University Hospitals, Cancer Institute, Altindag, Ankara, Turkey; 2 Department of Medical Oncology, Ankara University, Ankara, Turkey; 3 Department of Medical Oncology, Ankara Bilkent City Hospital, Ankara, Turkey; 4 Department of General Surgery, Division of Surgical Oncology, Faculty of Medicine, Ankara University, Ankara, Turkey; 5 Department of Medical Oncology, Ankara Etlik City Hospital, Ankara, Turkey

## Abstract

**Background:**

This study aimed to evaluate the demographic,” clinicopathologic, and prognostic characteristics of malignant peritoneal mesothelioma (MPeM), as well as the treatment options for the rare and heterogeneous MPeM population.

**Methods:**

A retrospective multi-center observational cohort study was conducted to evaluate patients with MPeM. Due to the heterogeneity of the study population, the study divided them into two main groups in terms of treatments, follow-up periods, and prognostic features. The first group comprised the patients who underwent cytoreductive surgery (CRS) and hyperthermic intraperitoneal chemotherapy (HIPEC), and the second group included the patients with metastatic disease for whom curative intent surgery was not possible. The patients’ diagnostic procedures and treatments were identified from medical records. Patients older than 18 years old were included in the study regardless of asbestos exposure. Well-differentiated papillary and multicystic mesothelioma histologic types were not included in the study.

**Results:**

The study evaluated 118 patients from five centers. Survival times, prognosis, and treatment responses were analyzed in both groups. The study showed that CRS-HIPEC was associated with longer overall survival (OS) and progression-free survival (PFS). Perioperative therapy was evaluated in subgroup analyses of this population and shown to provide survival benefits. The patients treated with chemotherapy (metastatic and medically inoperable patients and those for whom complete cytoreduction was not achievable) had a poorer prognosis than the surgery group. The study showed that life expectancy decreased significantly for the patients not suitable to undergo surgery for any reason.

**Conclusions:**

According to data from experienced centers, CRS-HIPEC is a treatment option recognized as effective, cost-effective, and safe, with better OS and PFS , as well as low morbidity and mortality rates similar to those in the literature. In addition, the platinum-pemetrexed combination continues to be an effective and acceptable treatment option for metastatic patients, those who are medically inoperable, and those for whom complete or near-complete cytoreduction is not achievable.

Diffuse malignant peritoneal mesothelioma (MPeM) is a rare, fatal tumor originating from the abdomen's peritoneal surfaces. Wynn and Miller reported MPeM for the first time in 1908.^1^ There are global demographic and epidemiologic differences in MpeM due to genetic variations, environmental and occupational exposure duration, onset time of exposure, and geographic variations.^2^

Usually, MPeM presents with non-specific symptoms, causing a delay in diagnosis until patients are at an advanced stage of disease. Patients present with symptoms such as abdominal pain, bloating, and distention, and have a higher symptom burden than patients with other types of cancer.^3^ Of all the mesotheliomas, MPeM is rarer than pleural mesothelioma and has a weaker relationship with asbestos. However, the most important known risk factor still is asbestos exposure.

The MPeM type accounts for 7–30 % of mesotheliomas.^2,4^ As with pleural mesothelioma, MPeM has different subtypes in terms of prognosis. Epithelioid is the main histologic type, with a better prognosis. Other types are biphasic and sarcomatoid MPeM.^5^ Most cases of MPeM can be detected by immunohistochemistry (IHC) and hematoxylin-eosin staining, which are indispensable parts of pathologic diagnosis. Specific biomarkers and IHC staining are essential in differentiating MPeM from adenocarcinoma or perıtoneal plasmacytoma.^2^ Positive staining of tumor-suppressor gene (WT1), calretinin, and D2-40 as IHC and negative staining with carcinoembryonic antigen (CEA), thyroid transcription factor 1 (TTF-1), and claudin-4 are necessary for MPeM confirmation.^6–8^ The protein associated with breast cancer-susceptibility gene 1 (BRCA1), another molecular marker also detected in IHC, is important in distinguishing malignant mesothelioma from reactive ones.^9,10^

Staging is essential in determining treatment options and prognosis. Because of differences in the distribution of intra-abdominal lymph nodes and the spread of extra-abdominal metastases, staging with classical tumor-node-metastasis (TNM) is not an accepted method. Therefore, Yan et al.^11^ introduced another staging system that evaluates the burden of disease in the abdominal region.

Usually, lesions are measured intraoperatively. This measurement is considered to be an accurate measure of peritoneal spread of tumor (T), intraperitoneal lymph node metastasis (N), and extraperitoneal metastasis (M). The Peritoneal Surface Oncology Group International (PSOGI) has divided T into four groups based on the peritoneal cancer index (PCI) as follows: T1 (PCI 1–10), T2 (PCI 11–20), T3 (PCI 21–30), and T4 (PCI 30–39), and the disease into three stages (stages 1, 2, and 3). Tumor burden and intra-abdominal spread are the most important prognostic factors for these patients, and they complicate staging. Staging methods include variations of radiologic and surgical staging systems. Intraoperative predictions or different imaging methods are used, but these methods may be significantly inconsistent with “true pathologic” evaluations. Therefore, these methods are not considered reliable and have not been standardized in clinical practice.^12^

This rare tumor, known to be fatal, has been associated with a high morbidity since it was identified. However, in recent years, longer survival has been achieved with some improvements in treatment**.** The survival time has significantly improved with the development of multimodal treatments such as combined systemic chemotherapies including cytoreductive surgery (CRS) and hyperthermic intraperitoneal chemotherapy (HIPEC).^13^

Some studies have demonstrated the effectiveness and importance of CRS-HIPEC as an initial treatment for suitable patients. Thus, with appropriate patient selection, the median overall survival (OS) of patients increased from less than 1 year to a period of 34 to 92 months.^11,14–16^ Patients with a high probability of achieving a complete or near-complete surgical response especially should be evaluated primarily for CRS-HIPEC. Clinical studies have shown that the patient's OS is prolonged if HIPEC is performed after an effective surgery.^17^ Because the success of resection is an important factor in prognosis, a cytoreduction score was defined.

Patients undergoing surgery also should be recorded using the completeness of cytoreduction score (CC) for residual disease after the procedure. The CC scores are as follows: CC-0 (no visible residual tumor) CC-11 (residual tumor <2.5 mm in diameter), CC-22 (residual tumor 2.5–2.5 cm in diameter), and CC-3 (residual tumor >2.5 cm in diameter or conglomerated lymph nodules present at any site).^18^ In the RENAPE study, the evaluation of 249 patients who underwent CRS HIPEC demonstrated that the group with lower CC scores (CC-0), epithelioid histology, and one drug versus two drugs had a longer survival.^19^

Despite the application of CRS-HIPEC for many patients, the chemotherapy agents are not standardized. The most commonly reported agents in the literature are mitomycin-c and cisplatin. The RENAPE trial evaluated different agents and combinations. The patients received cisplatin, cisplatin plus doxorubicin, mitomycin-c, oxaliplatin, or oxaliplatin with irinotecan and showed that combination chemotherapies also are associated with prolonged survival, especially for patients treated with platinum-based agents.^19,20^

For the CRS-HIPEC procedure, certain conditions such as the patient's performance status, gender, age, disease burden, possibility of complete or near-complete cytoreduction, histologic type, and presence of thrombocytosis should be evaluated.^15,21^ Many studies have shown that male gender, biphasic or sarcomatoid histology, age exceeding 60 years, deep tissue invasion, diffuse tumors in the mesentery and small intestine in perioperative imaging, and thrombocytosis adversely affect the possibility of complete cytoreduction. However, these conditions are associated with early recurrence and shorter survival in these studies.^15^ For patients who are unresectable, metastatic, medically inoperable, or incapable of achieving complete/near complete cytoreduction, systemic therapy should be considered. The preferred regimen usually is pemetrexed combined with cisplatin because its efficacy is known.^17^

Studies comparing the addition of cisplatin to single-agent pemetrexed have shown a longer progression-free survival (PFS; median, 8.7 vs 13.1 months). The response rate was 26 %, and the disease control rate was 71.2 %.^22,23^ Although the benefit for patients receiving adjuvant therapy is controversial, patients known to have poor prognostic features (sarcomatous or biphasic type, involvement of lymph nodes, Ki-67 >9 %, PCI >17) should be evaluated for adjuvant therapy after CRS-HIPEC.^2^

Besides conventional treatments, ongoing studies are focusing particularly on targeted therapies and immunotherapies as potential treatment options.

There are case-based studies suggesting that may be a targetable treatment for patients with MPeM with anaplastic lymphoma kinase (ALK) gene rearrangement. The effectiveness of alectinib is being monitored, especially in mesosthelioma patients with striatin (STRN) gene -ALK fusion, which is a rare condition.^24^ In a study by Raghav et al.^25^ investigating the efficacy of immunotherapies for patients with MPeM, 29 patients treated with immunotherapies had an objective response rate of 19 % and a 1-year OS of 68 % regardless of prior platinum-pemetrexed treatment.

In the review by Dietz et al.,^26^ various molecular alterations and potential targetable mutations were examined, and inhibitors of poly (ADP-ribose) polymerase (PARP), cyclin-dependent kinase 4/6 (CDK4/6), and enhancer of zeste homolog 2 (EZH2) were investigated. These options were discussed as potential alternatives for future therapies, and evidence supporting this view was presented.

## Patients and methods

This study evaluated the demographic and clinicopathologic characteristics, prognoses, and treatment responses of 118 patients with MPeM from five experienced centers. The study protocol complied with the tenets of the Helsinki Declaration and was approved by the ethics committee of Hacettepe University (approval no. 2023/08-07).

The median age of the patients was 62 years (interquartile range [IQR], 54–68.3 years). The patients comprised 52 women (44.1 %) and 66 men (55.9 %). The median age of the men was 60.9 years (range, 33–76 years), and the median age of the women was 63.5 years (range, 33–76 years). The basic patient characteristics are presented in Table 1.

**Table 1:** Baseline patient characteristics of the study population

Characteristics	Total 118 patients Frequency (%)
Age (years)	
Median (range)	62 (18–78)
<65	72 (31.9)
≥65	46 (26.1)
Gender	
Male	66 (55.9)
Female	52 (44.1)
Histologic type	
Epithelioid	84 (71.2)
Non-epithelioid	11 (9.3)
NOS	23 (19.5)
CC score (surgery group *n* = 46)	
CC 0–1 (group A)	31/46 (67,3)
CC 2–3 (group B)	15/46 (32.7)
PCI score (surgery group *n* = 46)	
PCI 1–20 (group 1)	29/46 (63)
PCI 21–39 (group 2)	17/46 (37)
Pretreatment ECOG	
0–1	74 (62.7)
2–4	44 (37.3)
Asbestos exposure	
Yes	61 (51.7)
No	46 (39)
Unknown	11 (9.3)
Ascites	
Yes	80 (67.8)
No	38 (32.2)
Treatments	
CRS-HIPEC	54 (45.8)
Medically inoperable and/or complete cytoreduction not achievable	37 (31.4)
Metastatic	12 (10.2)
No treatment	15 (12.7)

NOS, not otherwise specified; CC, completeness of cytoreduction score; PCI, peritoneal cancer index; ECOG, Eastern Cooperative Oncology Group; CRS, cytoreductive surgery; HIPEC, hyperthermic intraperitoneal chemotherapy

The patients represented different histologic subtypes with separate incidences and prognoses as follows: 84 patients were epithelioid; 11 patients were non-epitheloid (sarcomatoid, biphasic); and 23 patients had a subtype not otherwise specified (NOS). Varying amounts of ascites were present in 80 % of the patients, and asbestos exposure was present in 61 % of the patients ın the pretreatment period**.**

Various treatment options exist for peritoneal mesothelioma according to the spread of the disease. In terms of treatment, 54 patients underwent CRS and received HIPEC, 31 patients received adjuvant therapy after their prosedure, and 8 patients received neoadjuvant treatment. Cytotoxic chemotherapy was applied to 49 patients with metastatic disease (*n* = 12) and patients who were medically inoperable or incapable of complete cytoreduction (*n* = 37). The patients who were not candidates for curative-intent surgery had conditions such as sarcomatoid MPeM, extensive nodal metastases, extensive small bowel serosal or mesentery involvement, or similar conditions. Furthermore, 15 patients received no treatment due to poor Eastern Cooperative Oncology Group (ECOG) performance status (PS) comorbidities.

Some surgery-associated factors and their relationship with prognosis were evaluated in the surgical group. The peritoneal cancer index (PCI) was calculated for patients to determine tumor burden intraoperatively. The median PCI was 16 (IQR, 9–25), and the patients were grouped according to PCI score as follows: group 1 (0–20) and group 2 (21–39. Group 1 had better survival than group 2**.** Moreover, the CC scores of the patients were calculated after the procedure**.** The patients were divided into two groups according to the CC score as follows: group A (CC-0 and CC-1) and group B (CC-2 and CC-3). The majority of the patients received cisplatin/carboplatin-mitomycin-c as the HIPEC protocol**.** Mitomycin-carboplatin, gemcitabine-cisplatin, and oxaliplatin-based regimens alsowere used**.**

### Statistical Analysis

Continuous variables are presented as the median and IQR (25th–75th percentile). Categorical variables are presented as frequency and percentage. Progression-free survival was defined as the time from the initiation of the treatment to radiologic tumor progression or death from any cause. Overall survival was defined as the period from treatment initiation to the last follow-up visit and/or death. Kaplan–Meier was used for OS analyses. Survival of subgroups was compared with the log-rank test. The effect of variables on OS was measured by univariate Cox regression analysis. Variables significantly associated with OS in the univariate Cox regression analysis were evaluated with multivariate modeling. The Cox proportional hazards model was used to calculate the hazard ratio (HR) and corresponding 95 % confidence interval (CI). All statistical tests were two-tailed, and a *p* value lower than 0.05 was considered statistically significant. The statistical analyses were performed with SPSS, version 25.0 (IBM Inc., Armonk, NY, USA).

## Results

**Fig. 1:**
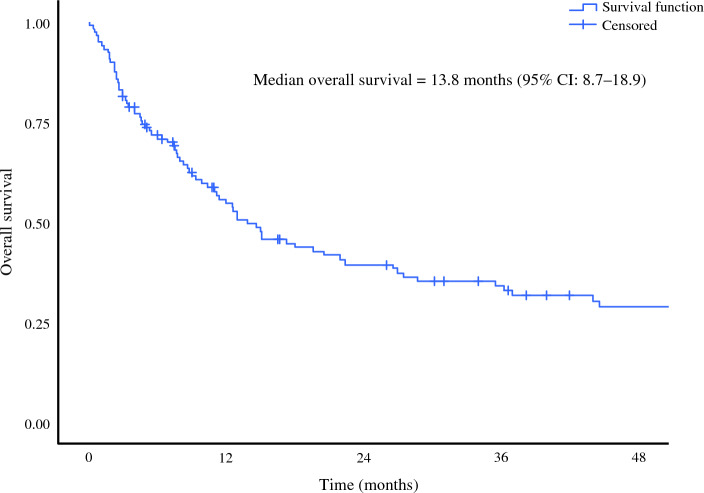
Kaplan-Meier curves for overall survival (OS) of patients with malignant peritoneal mesothelioma.

The median OS was 13.8 months (95 % CI, 8.7–18.9 months), and the median follow-up time was 10.9 months in the overall population (IQR: 4–35.6). In the overall population, the 1-year OS was 54 %, and the 2-year OS was 39 % (Fig. 1).

In our study, we aimed to perform a comprehensive analysis of treatments. Log-rank analysis was used to compare groups that were medically inoperable or incapable of complete cytoreduction, metastatic, or treated with CRS-HIPEC. The median overall survivals were respectively 12.6, 11.2, and 44.6 months and statistically significant (*p* < 0.001). The analysis did not include patients who could not be treated due to comorbidities or poor performance scores.

### CRS and HIPEC Population

**Fig. 2:**
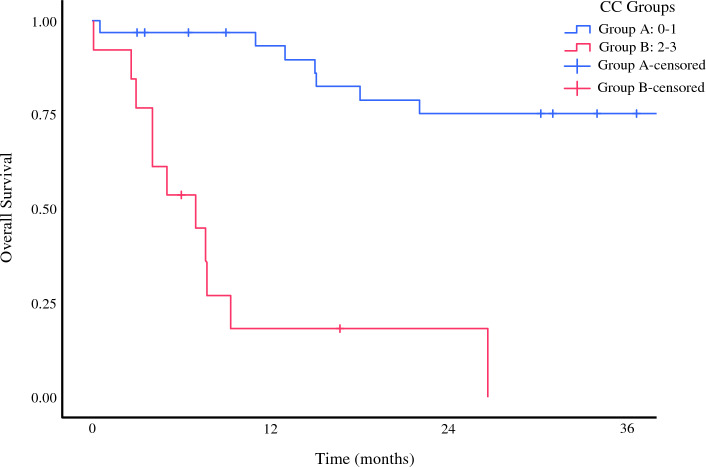
Kaplan-Meier curve for survival according to CC score and 1-year survial rates of 93 % for group A and 17 % for group B. CC, completeness of cytoreduction

**Fig. 3:**
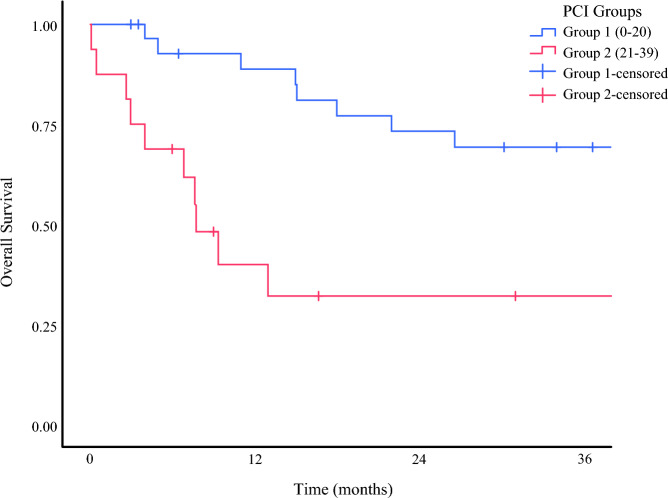
Kaplan-Meier curve for survival according to PCI score and 1-year survial rates of 88 % for group 1 and 32 % for group 2

The study evaluated 54 patients who underwent CRS-HIPEC in experienced centers. The median OS was 44.6 months (95 % CI, 0–102 months), and the median PFS was 7.63 months (95 % CI, 4,45–10.8 months). Of the patients in this group, 25 % received second- and third-line post-progression therapy**.** Early mortality occurred for two patients due to postoperative complications. Earlier, it was mentioned that the study had two patient groups (groups A and B) according to the CC score. We compared the survival rates of these two groups. For this population, data on the CC and PCI scores of 46 patients could be accessed. Groups A and B were compared according to the CC score. The group with the low CC score had a significantly better survival, with a 1-year survival rate of 93 % compared with 17 % (Fig. 2). Similarly, we categorized the PCI scores into groups 1 and 2. The group with the lower PCI score had better survival, with a 1-year survival rate of 88 % compared with 32 % (Fig. 3).

The adjuvant and neoadjuvant subgroups were evaluated to determine the effectiveness of perioperative treatment with CRS-HIPEC. Four to six cycles of adjuvant or neoadjuvant treatment, mostly consisting of platinum and pemetrexed, were administered. The patients who preferred perioperative treatment had a high tumor burden, a high CC score, a high PCI score, and non-epithelioid histology. The perioperative treatment group had a longer OS than the group that had CRS-HIPEC alone. Although it did not reach statistical significance, there was a 23-month difference compared with adjuvant treatment. (67 months [95 % CI, 0–99 months] vs 44 months [95 % CI, 0–134 months; *p* = 0.596). In addition, a small group of eight patients who received neoadjuvant therapy had better CC scores and survival time. Because the number of patients in the neoadjuvant group was limited, OS and PFS could not be calculated. More patients and prospective studies are needed to clearly demonstrate the importance of perioperative treatment.

Age, gender, ascites, histologic type, CC score, PCI score, and asbestos exposure were evaluated with univariate analysis for CRS-HIPEC population (Table 2). The Cox regression analysis did not include ECOG PS for this group because the performance status of the patients was 0 or 1, and for the majority was 0, but it was included in the analysis for the non-surgical group.

**Table 2:** Uni- and multivariate analyses for the CRS-HIPEC population

Variable	Univariable analysis			Multivariable analysis		
	HR	95 % CI	*p* value	HR	95 % CI	*p* value
Age (years) *<*65 vs ≥65	1.31	0.58–2.95	0.522			
Gender Male vs female	0.695	0.326–1.482	0.347			
CC score (0–1/2–3)	11.17	4.03–30.9	**<0.001**	**8.839**	2.901–26.937	**<0.001**
PCI score Group 1–2 (0–20)/(21–39)	3.541	1.522–8.236	**0.005**	1.807	0.674–4.847	0.24
Histologic type Epithelioid/(non-epitheloid and NOS)	0.399	0.94–1.69	0.156			
Ascites Yes/no	1.057	0.495–2.258	0.886			
Asbestos exposure No Yes	0.99	0.406–2.41	0.982			

CRS, cytoreductive surgery; HIPEC, hyperthermic intraperitoneal chemotherapy; HR, hazard ratio; CI, confidence interval; CC completeness of cytoreduction score; PCI, peritoneal cancer index; NOS, not otherwise specified

Univariate analysis was performed with these parameters: high CC score (CC-2 or CC-3) (HR, 11.1; 95 % Cl, 4.03–30.9; *p* < 0.001), and high PCI score (HR, 3.541; 95 % Cl, 1.522–8.236; *p* = 0.005) were associated with poor survival outcomes, but there was no significant relationship between age, histologic type, gender, and asbestos exposure in the survival analysis.

Statistically significant parameters in the univariate analysis were included in the in multivariate analysis. The multivariate analysis showed that the CC score was the most important independent prognostic factor. (HR, 8.839; 95 % Cl, 2.901–26.937; *p* < 0.001). As a result, optimal surgery and complete or near-complete surgery were associated with longer survival.

### Non-Surgical Population

The non-surgical population comprised patients were metastatic, medically inoperable, or incapable of complete cytoreduction. These conditions were evaluated separately. The results showed that metastatic disease had a poor prognosis. However, if patients were not candidates for surgery or HIPEC, they had poor outcomes, especially with high disease burden and nodal disease such as metastatic disease.

For this non-surgery (non-metastatic) group, the median OS was 12.6 months (95 % CI, 9.17–16.03 months), and the median PFS was 5 months (95 % Cl, 2.074–7.926 months) with first-line treatment. The majority of the patients in this group received a cisplatin-pemetrexed combination.

For the metastatic group, the median OS was 11.2 months (95 % CI, 6.014–16.38 months), and the median PFS was 2.2 months (95 % Cl, 0–4.897 months) with first-line treatment. The patients in both groups received at least one line of treatment and an average of four chemotherapy cycles.

No treatment was administered to 15 patients due to poor performance and comorbidities. This group had a very poor median survival of 1.7 months (95 % CI, 1.09–2.44 months).

Similarly, age, gender, ECOG PS, ascites, histologic type, and asbestos exposure were evaluated with univariate analysis (Table 3). Poor survival outcomes were associated with a pretreatment poor performance score (ECOG PS 2–4: HR, 2.478; 95 % Cl, 1.296–4.738; *p* = 0.007) and presence of ascites (HR, 2.911; 95 % Cl, 1.31–6.467; *p* = 0.004), but there was no significant relationship of age, histologic type, gender, and asbestos exposure to survival analysis.

**Table 3:** Uni- and multivariate analyses for the non-surgical group

Variable	Univariable analysis			Multivariable analysis		
	HR	95 % CI	*p* Value	HR	95 % CI	*p* Value
Age (years) *<*65 vs ≥65	1.601	0.851–3.011	0.150			
Gender Male vs female	0.693	0.366–1.312	0.265			
Pretreatment ECOG PS 0–1 vs *>*1	2.478	1.296–4.738	**0.007**	5.309	2.377–11.86	**<0.001**
Histologic type Epithelioid/(non-epitheloid and NOS)	0.645	0.340–1.223	0.176			
Ascites Yes/no	2.911	1.31–6.467	**0.004**	5.99	2.386–15.037	**<0.001**
Asbestos exposure No Yes	0.831	0.429–1.612	0.582			

HR, hazard ratio; CI, confidence interval; ECOG, Eastern Cooperative Oncology Group; PS, performance status; NOS, not otherwise specified

Statistically significant parameters in univariate analysis were used in the multivariate analysis**.** The multivariate analysis showed that poor performance status (HR, 5.309; 95 % Cl, 2.377–11.86; *p* < 0.001) and presence of ascites (HR, 5.99; 95 % Cl, 2.386–15.037; *p* < 0.001) were independently associated with poor survival outcomes**.**

## Discussion

Malignant peritoneal mesothelioma continues to be a difficult disease to manage and treat. Spread of the disease, comprehensive patient evaluation for appropriate treatment options, histopathologic characteristics, and the surgeon's experience are important for prognosis, and all of these factors influence the choice of treatment method.

There is no clear consensus because of the low incidence, lack of prospective studies, and the respective nature of the majority of the studies based on case reports. However, it seems recently that experienced centers have reached a consensus on approaches such as CRS-HIPEC. Therefore, this study aimed to analyze the safety and effectiveness of treatment options such as CRS-HIPEC, perioperative treatment, and cytotoxic chemotherapies in non-surgical groups.

Studies showed that the clinicopathologic features and treatment options for this rare tumor group are heterogeneous and varied. The effectiveness of treatments and the survival rates vary widely between methods and centers. The key point is to apply the most beneficial treatment to the patients.

When making a decision about cytoreduction, cliniciants must accurately determine whether the patient is a candidate for complete resection because the best survival is seen with a low CC score. If cytoreduction is performed for an unsuitable patient, the treatment will not benefit survival, and the patient will be exposed to a procedure with high morbidity and mortality**.** Furthermore, as emphasized previously, perioperative treatments are another approach expected to improve results in addition to appropriate surgery. Similar to our study, a study conducted by Chatterjee et al.,^27^ which included 115 patients, showed that adjuvant chemotherapy improved OS. However, the survival benefit of neoadjuvant therapy before surgery has not been demonstrated.

Cytotoxic chemotherapies are still the best option for patients who are not candidates for curative surgery. If the patient is not suitable for surgery, we apply chemotherapy, and we can provide more benefits. Otherwise, a high CC score after surgery can cause detrimental effects.

Whereas the OS of the non-metastatic group was approximately 1 year with conventional treatments, CRS-HIPEC provided a significant benefit, extending survival to about 44 months in our study population. Literature data also supported our survival rates, with median survival data of 34 to 92 months.^11,14–16^ Especially in the last decade, as the experiences with CRS-HIPEC have increased, historical approaches have been abandoned, and it has started to be one of the main treatment options, especially for well-selected patients.

However, success depends on an experienced center and an experienced surgeon. Otherwise, morbidity can reach 30 %, and the perioperative mortality rate can be as high as 2 %.^28,29^

In the review written by Sugarbaker,^30^ 1 % mortality was reported. In our study, the first-month period has been considered as early mortality, and the rate has been found to be 1.8 %.

In addition to the importance of an optimum surgical procedure, we reviewed the efficacy of chemotherapy in non-surgical groups. İn 2003, Vogelzang et al.^31^ demonstrated for the first time that pemetrexed-cisplatin is an effective regimen. In their study, a median OS of 12.1 months was demonstrated with the combination of pemetrexed and cisplatin, compared with 9.3 months when cisplatin alone was used. Additionally, the progression-free survivals were 5.7 months and 3.9 months, respectively.

In another study conducted by Nagata et al.,^17^ the efficacy of pemetrexed plus cisplatin as first-line treatment for unresectable patients was demonstrated by a median PFS of 7.1 months and a median OS of 15.4 months. In our study, most of the patients had received platinum-pemetrexed and had a median survival time was 12.5 months, whereas for other combinations or the single-agent treatment population, the median survival time was 7.9 months.

The limitations of our study were its retrospective nature, the heterogeneity of the patient group, and the differences in patient selection between surgical centers and other methods. However, many studies are retrospective and single-center investigations, and due to the rare occurrence of MPeM, evidence from randomized clinical trials regarding treatment and patient management is insufficient. In this respect, our study is important regarding patient management. Another limitation of our study was the inadequate number of patients and short follow-up duration for perioperative treatments, which are crucial for MPeM. Although not statistically calculated, the results of perioperative treatment for a limited number of patients appear promising.

## Conclusions and future developments

Areas still exist that are not fully understood, particularly in relation to the treatments for this uncommon disease. It is important that the decision for a patient’s treatment be discussed with a team of experts from different fields to determine which treatment will offer the most benefits.

Of course, if an experienced surgeon anticipates achieving complete or nearly complete cytoreduction, the recommended course of action is surgery.

Another large population that cannot undergo surgery for medical reasons or achieve a complete cytoreduction through surgery can still benefit from treatment that combines pemetrexed with platinum. Clinical studies have proven the effectiveness of this treatment, making it a viable option.

As mentioned in this report, both immunotherapies as well as various molecular alterations and potential targetable mutations (e.g., poly [ADP-ribose] polymerase [PARP], cyclin-dependent kinase 4/6 [CDK4/6], enhancer of zeste homolog 2 [EZH2], anaplastic lymphoma kinase [ALK]) continue to be investigated as potential alternatives for future treatments and hold promise.

Therefore, as previously mentioned, determining the appropriate treatment for each patient can significantly increase survival time. It is known that peritoneal mesothelioma starts and progresses with non-specific symptoms, causing delays in diagnosis and depriving the patient of a very effective treatment. Therefore, in case of clinical suspicion, especially for patients from endemic regions, those with occupational exposure, and those with early onset exposure, advanced investigations or screening programs should be developed to prevent delays in diagnosis.
